# The function of peroxisome proliferator-activated receptors PPAR-γ and PPAR-δ in *Mycobacterium leprae*-induced foam cell formation in host macrophages

**DOI:** 10.1371/journal.pntd.0008850

**Published:** 2020-10-19

**Authors:** Yuqian Luo, Kazunari Tanigawa, Akira Kawashima, Yuko Ishido, Norihisa Ishii, Koichi Suzuki

**Affiliations:** 1 Department of Clinical Laboratory Science, Faculty of Medical Technology, Teikyo University, Tokyo, Japan; 2 Department of Laboratory Medicine, Nanjing Drum Tower Hospital, Nanjing University Medical School, Nanjing, China; 3 Department of Molecular Pharmaceutics, Faculty of Pharma-Science, Teikyo University, Tokyo, Japan; 4 Leprosy Research Center, National Institute of Infectious Diseases, Tokyo, Japan; Fundação de Medicina Tropical Doutor Heitor Vieira Dourado, BRAZIL

## Abstract

Leprosy is a chronic infectious disease caused by *Mycobacterium leprae* (*M*. *leprae*). In lepromatous leprosy (LL), skin macrophages, harboring extensive bacterial multiplication, gain a distinctive foamy appearance due to increased intracellular lipid load. To determine the mechanism by which *M*. *leprae* modifies the lipid homeostasis in host cells, an *in vitro M*. *leprae* infection system, using human macrophage precursor THP-1 cells and *M*. *leprae* prepared from the footpads of nude mice, was employed. RNA extracted from skin smear samples of patients was used to investigate host gene expressions before and after multidrug therapy (MDT). We found that a cluster of peroxisome proliferator-activated receptor (PPAR) target genes associated with adipocyte differentiation were strongly induced in *M*. *leprae-*infected THP-1 cells, with increased intracellular lipid accumulation. PPAR-δ and PPAR-γ expressions were induced by *M*. *leprae* infection in a bacterial load-dependent manner, and their proteins underwent nuclear translocalization after infection, indicating activation of PPAR signaling in host cells. Either PPAR-δ or PPAR-γ antagonist abolished the effect of *M*. *leprae* to modify host gene expressions and inhibited intracellular lipid accumulation in host cells. *M*. *leprae*-specific gene expressions were detected in the skin smear samples both before and after MDT, whereas PPAR target gene expressions were dramatically diminished after MDT. These results suggest that *M*. *leprae* infection activates host PPAR signaling to induce an array of adipocyte differentiation-associated genes, leading to accumulation of intracellular lipids to accommodate *M*. *leprae* parasitization. Certain PPAR target genes in skin lesions may serve as biomarkers for monitoring treatment efficacy.

## Author summary

Leprosy is a chronic infectious disease caused by *Mycobacterium leprae* (*M*. *leprae*). Lipid-enriched intracellular environment is important for the parasitization of *M*. *leprae*. During anti-leprosy treatment, chemotherapy-killed bacilli can remain in host tissues for a long time, making it difficult to determine the treatment efficacy by Zeihl-Nelson’s staining-based bacterial index (BI) test. In this study, we found that host peroxisome proliferator-activated receptor (PPAR) signaling is responsible for modification of intracellular lipid homeostasis to accommodate *M*. *leprae* parasitization in host macrophages. In skin smear samples of patients, *M*. *leprae*-derived gene expressions were detected both before and after anti-leprosy treatment, whereas human PPAR target gene expressions were dramatically diminished after the treatment. These results further our understanding of *M*. *leprae* intracellular parasitization, and suggest that PPAR signaling may be a novel therapeutic target for treating *M*. *leprae* infection and monitoring the expressions of certain PPAR target genes in skin lesions may be helpful to evaluate the treatment efficacy and recurrent infection.

## Introduction

Leprosy is an ancient chronic infectious disease caused by *M*. *leprae*, an indolent-growing obligate intracellular bacterial pathogen. Despite the success of multidrug therapy (MDT) that has reduced the leprosy burden over years, this disease remains an important cause of morbidity in many developing countries, with over 200,000 new cases reported worldwide annually [1]. During the course of MDT, an acute aggravating episode known as lepra reaction, which is putatively triggered by an intense immune response to the chemotherapy-uncovered bacilli antigens, can occur and may cause severe and irreversible nerve damage [2]. MDT-killed bacilli can remain in host tissues for a long time, which can be detected in Zeihl-Nelson’s staining-based bacterial index (BI) test. Thus, BI drops very slowly during the treatment, and sometimes remains unchanged even after completion of 12-month MDT, making it difficult to determine the drug efficacy or relapse of active infection. Therefore, it is still important to explore new approaches to control infection and to evaluate host response to anti-leprosy treatment.

Leprosy manifests as a spectrum of clinical forms dictated by the magnitude of host immune response mounted against the *M*. *leprae* infection. Lepromatous leprosy (LL) is at the severer extreme, characterized by widespread skin lesions harboring extensive bacterial multiplication [3]. The *M*. *leprae*-packed histiocytes, typically macrophages, gain a distinctive foamy appearance due to a large amount of cytoplasmic lipid accumulation, hence named foam cells [3]. Close examination of these foam cells revealed that *M*. *leprae* resides and replicates within enlarged, lipid-filled phagosomes [4], suggesting significant modifications in host lipid metabolism adapted to *M*. *leprae* infection. Lipid-rich environment is believed to be critical for the intracellular parasitization of *M*. *leprae*, putatively via providing the bacteria with nutrients and sheltering in the granuloma environment [5]. However, the mechanism that modifies the lipid homeostasis in *M*. *leprae-*infected host cells remains largely unclear.

Peroxisome proliferator-activated receptors (PPARs), including PPAR-α, PPAR-δ, and PPAR-γ, are a family of ligand-activated nuclear receptors that function as transcription factors to regulate gene expressions closely related to lipogenesis, lipid metabolism, and foam cell formation in macrophages [6]. The implication of PPARs in mycobacterial infections such as infection by *M*. *bovis* bacillus Calmette-Guerin (BCG) or *M*. *tuberculosis* through regulating lipid influx/efflux and lipid droplet formation in host macrophages began to gain recognition [7–9]. Interference with PPAR signaling was shown to result in decreased intracellular lipid accumulation and increased *Mycobacterium* killing in *M*. *tuberculosis*-infected macrophages *in vitro* [8]. To date, however, it was not clear whether activation of PPAR signaling has dictated the alteration of host lipid homeostasis in *M*. *leprae* infection.

## Methods

### Ethic statement

Human slit-skin samples were used according to the guidelines approved by the Ethical Committee of the National Institute of Infectious Disease (Tokyo, Japan) and Teikyo University (Tokyo, Japan). All samples were anonymized before use. Skin smear samples were obtained by using the same protocol as that used for BI test with written informed consent. Briefly, the skin at the smear sites was sterilized with a cotton wad drenched in alcohol and air-dried. An incision approximately 5 mm x 2 mm in the skin was made using a new stainless-steel blade (Feather Safety Razor, Osaka, Japan) which was put on a scalpel handle, while pinching the incision to make sure the it remains bloodless. Skin tissue fluid and pulp were collected by scraping inside the cut once or twice with the blade. The material scraped from the incision in the blade was rinsed in 1 ml of sterile 70% ethanol and stored at 4°C before RNA purification.

### Cell culture, infection with *M*. *leprae*, and treatment with antagonists or agonists of PPARs

THP-1, a human promonocytic cell line, was obtained from the American Type Culture Collection (ATCC; Manassas, VA). Cells were cultured in 10 cm tissue dishes in RPMI medium supplemented with 10% charcoal-treated fetal bovine serum, 2% nonessential amino acids, and 50 mg ml^-1^ penicillin/streptomycin at 37°C in 5% CO_2_. *M*. *leprae* was prepared from the footpads of nude mice as previously described [10,11]. Live or heat-killed (80°C, 30 min) bacilli were added to cells, at typically multiplicity of infect (MOI) = 100 or otherwise indicated. Cells were further cultured for RNA or protein purification. GSK3787, BADGE, L-164,041, and dimethyl sulfoxide (DMSO) were purchased from Sigma Aldrich (Saint Louis, MO). GSK3787, BADGE and L-164,041 were dissolved in DMSO. Stocking solutions were diluted in culture medium by 10,000-fold to indicated working concentrations, and their treatment began 2 h prior to *M*. *leprae* infection.

### RNA isolation, reverse transcription (RT)-PCR, quantitative real-time PCR, and touchdown PCR

RNA was prepared from cultured cells using the RNeasy Mini Kit (Qiagen Inc., Valencia, CA). RNA was extracted from slit-skin smear specimens as previously described [10]. Slit-skin smear specimens that were stored in 1 ml of sterile 70% ethanol were centrifuged at max speed for 1 min at 4°C. RNA was then isolated from the retained pellets with RNeasy Mini Kit (Qiagen, Hilden, Germany), using the same protocol as that used for cultured cells. RNA was eluted in 20 μl of elution buffer. RNA concentration and purity were assessed using a Genequant Pro Spectrophotometer (GE Healthcare UK Ltd, Buckinghamshire, UK), and RT-PCR was performed using the High-Capacity cDNA Reverse Transcription Kit (Applied Biosystems, Foster City, CA). Real-time PCR was performed using Fast SYBR Green Master Mix (Applied Biosystems) and the StepOnePlus Real-Time PCR System (Applied Biosystems) according to the manufacturer’s instructions. Relative mRNA expression levels were normalized against corresponding β-ACTIN levels. Touchdown PCR was performed using a Thermal Cycler Dice (Takara Bio, Tokyo, Japan). Briefly, the PCR mixture was first denatured for 5 min at 94°C, followed by 20 cycles of three-temperature PCR consisting of denaturation for 30 sec at 94°C, annealing for 30 sec that started at 65°C and decreased 0.5°C every cycle to 55°C, and extension at 72°C for 45 sec. An additional 30 cycles were performed with a fixed annealing temperature of 55°C. The touchdown PCR products were analyzed by 2% agarose gel electrophoresis. The sequences of PCR primers were as listed in S1 Table.

### Protein preparation and Western blot analysis

Cells were lysed in a lysis buffer containing 50 mM HEPES, 150 mM NaCl, 5 mM EDTA, 0.1% NP40, 20% glycerol and a cOmplete Mini protease inhibitor cocktail tablet (Roche Diagnostics, Basel, Switzerland) for 1 h. The lysates were centrifuged at max speed at 4°C for 20 min to recover cell proteins. Protein concentration was determined using DC protein assay reagents (BIO-RAD, Hercules, CA) and a VMax Kinetic Microplate Reader (Molecular Devices, Sunnyvale, CA) according to the manufacturer’s instructions. Proteins were separated on NuPage 4%–12% Bis-Tris gels (Invitrogen) and transferred to polyvinylidene fluoride (PVDF) membranes using Novex iBlot PVDF transfer stacks (Life Technologies, Waltham, MA). The membranes were washed with PBS containing 0.1% Tween 20 (PBST), blocked with PBST containing 5% nonfat milk for 1 h, and then incubated overnight at 4°C with a rabbit anti-PPAR-δ antibody (ab8937, Abcam, Cambridge, UK; 1:5000) or a rabbit anti-PPAR-γ antibody (#2435, Cell Signaling Technology, Danvers, MA; 1:5000). After washing with PBST, membranes were incubated with a biotin-conjugated donkey anti-rabbit IgG antibody (GE Healthcare; 1:20,000) for 1 h, washed with PBST, and then incubated with streptavidin horseradish peroxidase (GE Healthcare; 1:20,000) for 1 h. Specific bands were visualized using Immunostar LD reagent (Wako Pure Chemical, Osaka, Japan) and captured with a C-DiGit blot scanner (LI-COR, Lincoln, NE) according to the manufacturer’s instructions.

### Oil red O staining

THP-1 cells grown on poly-L-lysine coated culture coverslips (Matsunami Glass, Osaka, Japan) in a 24-well plate were infected with *M*. *leprae* for 48 h. THP-1 cells were fixed in 10% formalin for 10 min and then washed with Dulbecco’s PBS (DPBS) and balanced with 60% isopropanol for 1 min before staining with oil red O (Muto Pure Chemicals, Tokyo, Japan) for 10 min. The cells were counterstained with hematoxylin for 5 min followed by ethanol dehydration and coverslip sealing. Images of all the oil Red O staining were captured using a digital camera attached to the light microscope and analyzed using the image analysis software ImageJ. Positive-labeling (red) was defined by the application of a color threshold mask, and the same threshold was applied to all sections. The lipid droplet area sizes were normalized by the control group as indicated.

### Immunofluorescence staining

Cells grown on poly-L-lysine coated culture coverslips (Matsunami Glass, Osaka, Japan) in a 24-well plate were infected with FITC-conjugated *M*. *leprae* for 48 h. After discard of the supernatants, cells were washed with PBS 5 times to remove excess extracellular *M*. *leprae*, fixed with 10% buffered formalin (Wako Pure Chemical) for 15 min, permeabilized with 0.3% Triton X-100 (Wako Pure Chemical) in PBS for 5 min, and blocked with 0.5% bovine serum albumin (BSA) (Sigma Aldrich) in PBS for 1 h. Immunofluorescence staining was performed by incubating the coverslips with a rabbit anti-PPAR-δ antibody (ab8937, Abcam; 1:500) or a rabbit anti-PPAR-γ antibody (#2435, Cell Signaling Technology; 1:500) in PBS at 4°C overnight. After washing with PBS, coverslips were then incubated with a mixture of Alexa Fluor 594-conjugated chicken anti-rabbit IgG antibody (Life Technologies; 1:1,000) for 1 h at room temperature. The nuclei were counterstained with Hoechst 33258 (Life Technologies; 1:1,000) for 3 min at room temperature. Cover slips were placed on a piece of glass slide with fluorescence mounting medium (Dako, Tokyo, Japan). Immunofluorescence was visualized and the images were captured with an FV10i-LIV laser scanning microscope (Olympus, Tokyo, Japan).

### Statistical analysis

All experiments were repeated at least three times with different batches of cells, and the mean ± SD of these experiments was calculated. The significance of the differences between experimental values was determined by an unpaired two-tailed t-test where *p* < 0.05 was significant.

## Results

### Induction of PPARs target gene expressions associated with adipocyte differentiation in *M*. *leprae*-infected foam cells

PPARs regulate the expression of genes involved in lipid droplet formation, lipid transportation and uptake, and intracellular lipid storage, such as adipose differentiation-related protein (*ADRP*) [12], fatty acid-binding protein 4 (*FABP4*) [13–15], scavenger receptor *CD36* [16–18], apolipoproteins (*APOE*, *APOC*) [19], acyl-CoA synthetase long chain family (*ACSL*) [20,21]. Nuclear PPARs bind to the peroxisome proliferators response elements (PPREs) in the promoters of the above-mentioned target genes to initiate transcription, eventually leading to a differentiated adipocyte phenotype [12].

**Fig 1:**
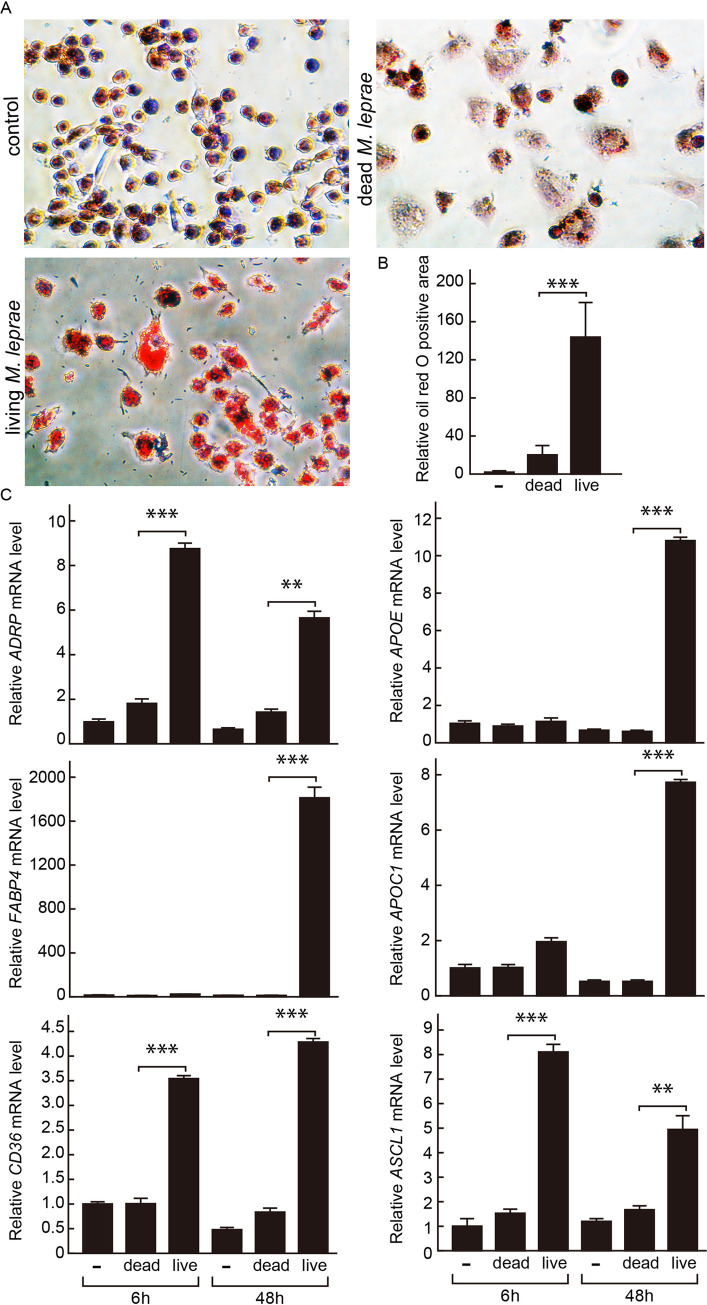
Adipocyte differentiation-associated genes were induced by *M*. *leprae* infection in parallel with foam cell formation in THP-1 cells. **(**A) THP-1 cells grown on glass coverslips in 24-well plates were infected with *M*. *leprae* (MOI = 100) or stimulated with heat-killed *M*. *leprae* for 48 h, followed by oil red O staining and hematoxylin counter staining. (B) Oil red O positive area of 100 cells were quantified using ImageJ software and normalized against that of non-infected control cells. Data are presented as mean ± SD relative to the control cells (n > 7). ***P < 0.001, compared to dead *M*. *leprae-*stimulated cells. (C) Total RNAs were extracted from THP-1 cells infected with *M*. *leprae* (MOI = 100) or stimulated with heat-killed *M*. *leprae* at 0, 6, and 48 h. Relative mRNA levels of *ADRP*, *FABP4*, *CD36*, *APOE*, *APOC1*, and *ASCL1* were evaluated by real-time PCR. Data are presented as mean ± SD relative to the control cells (n = 3). **P < 0.01; ***P < 0.001, compared to dead *M*. *leprae-*stimulated cells.

To evaluate potential activation of PPAR signaling in *M*. *leprae-*infected cells, we first checked the expression levels of PPAR target genes following *M*. *leprae* infection. Mimicking the characteristic histological features seen in LL lesions, THP-1 cells infected with *M*. *leprae* (MOI = 100) accumulated a large amount of intracellular lipid droplets within 48 h, as demonstrated by oil red O staining (Fig 1A). In contrast, heat-killed *M*. *leprae* was much less capable to induce such foam cell formation in THP-1 cells (Fig 1A and 1B). The mRNA levels of PPAR target genes associated with adipocyte differentiation, including *ADRP*, *FABP4*, *CD36*, *APOE*, *APOC1*, *ASCL1*, were strongly induced in THP-1 cells at 6 or/and 48 h after infection by *M*. *leprae* but not stimulation by dead *M*. *leprae*, as showed by real-time PCR (Fig 1C).

### Gene and proteins expressions of PPAR-δ and PPAR-γ were significantly increased in *M*. *leprae*-infected foam cells

**Fig 2:**
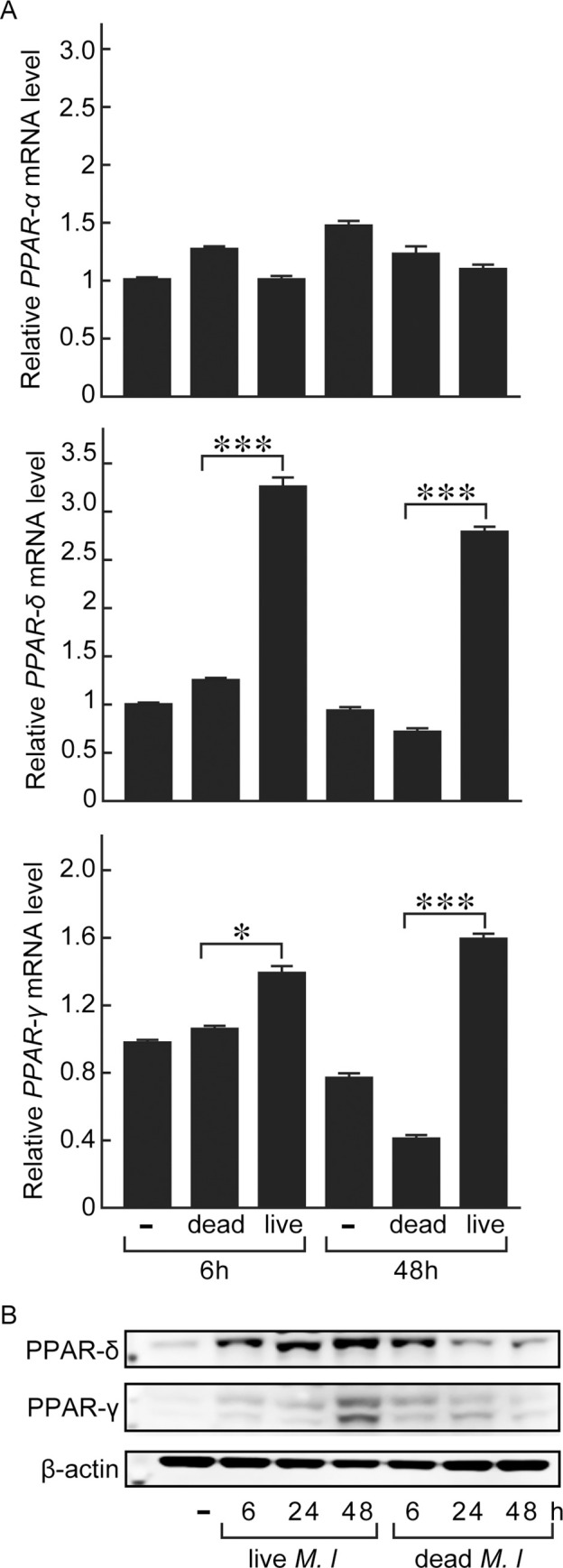
PPAR-δ and PPAR-γ mRNA and protein expressions were significantly induced in *M*. *leprae*-infected foam cells. THP-1 cells were infected with *M*. *leprae* (MOI = 100) or stimulated with heat-killed *M*. *leprae*. Cellular mRNAs and proteins were extracted at 0, 6, and 48 h after infection/stimulation. (A) PPAR-α, PPAR-δ, and PPAR-γ mRNA levels were evaluated by real-time PCR. Data are presented as mean ± SD relative to the control cells (n = 3). *P < 0.05; ***P < 0.001, compared to dead *M*. *leprae-*stimulated cells. (B) PPAR-δ and PPAR-γ protein levels were evaluated by Western blotting.

**Fig 3:**
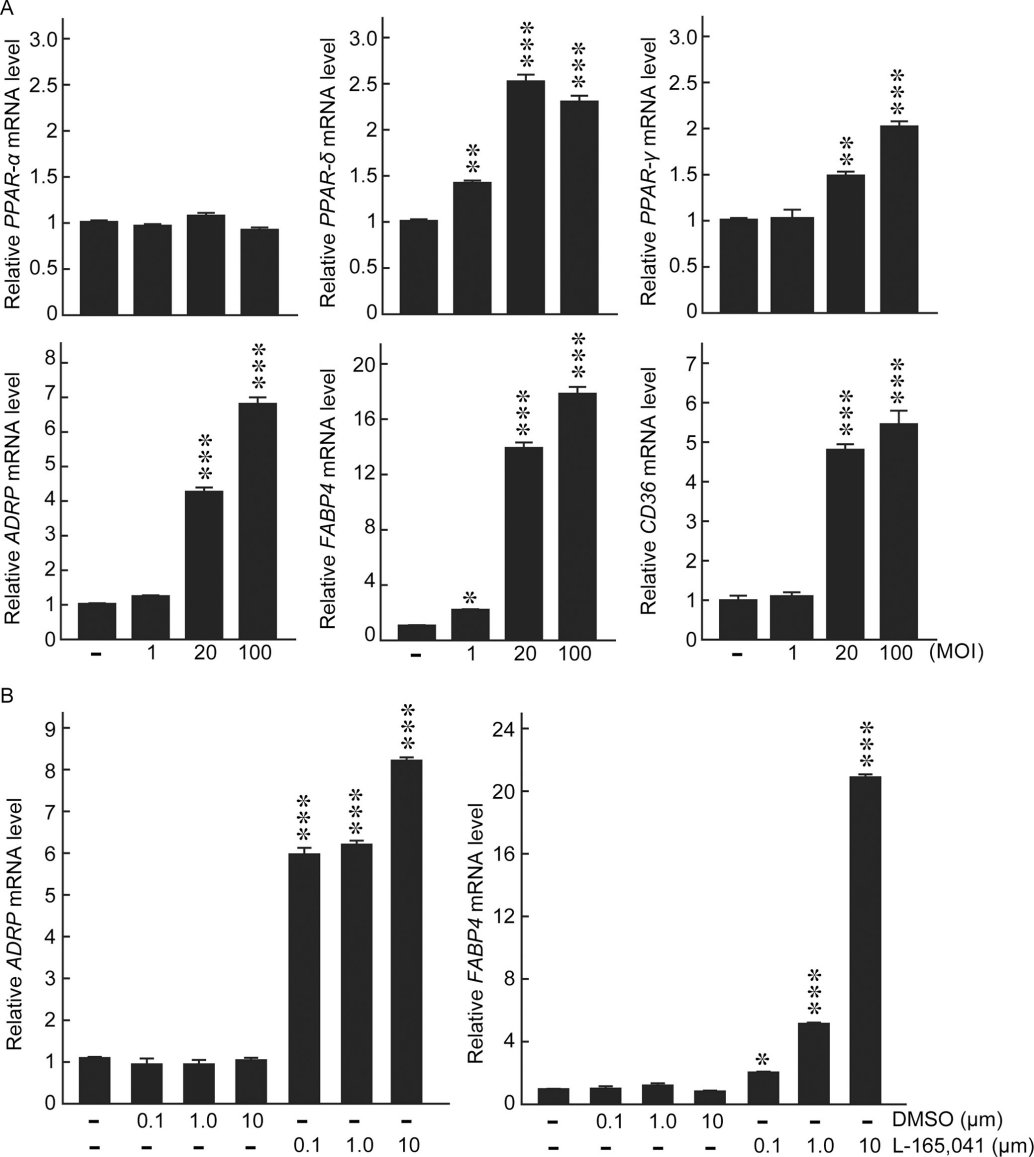
Adipocyte differentiation-associated genes were induced by *M*. *leprae* infection or PPAR-δ agonist L-165,041 in does-dependent manners. (A) THP-1 cells were infected with *M*. *leprae* at indicated MOI for 48 h. Total RNAs were extracted, relative mRNA levels of *PPAR-α*, *PPAR-δ*, *PPAR-γ*, *ADRP*, *FABP4*, and *CD36* were evaluated by real-time PCR. (B) THP-1 cells were incubated in medium containing L-165,041 or vehicle DMSO at indicated concentrations. Total RNAs were extracted, relative mRNA levels of *ADRP* and *FABP4* were evaluated by real-time PCR. Data are presented as mean ± SD relative to the control cells (n = 3). *P < 0.05; **P < 0.01; ***P < 0.001, compared to control cells.

The three major subtypes in PPAR superfamily: PPAR-α, PPAR-δ, and PPAR-γ, presumably act cooperatively to leverage the balance of intracellular lipid homeostasis as they share a good number of target genes associated with adipocyte differentiation [12]. To dissect the participation of each subtypes in *M*. *leprae*-infected host cells, we examined the expression levels of PPAR subtypes in response to *M*. *leprae* infection in THP-1 cells. PPAR-α gene expression was rather inert to *M*. *leprae* infection (MOI = 100), whereas the gene expressions of PPAR-δ and PPAR-γ were specifically induced by *M*. *leprae* infection but not stimulation with dead *M*. *leprae* (Fig 2A). In accordance with the gene expression levels, sustainable increases in the protein expressions of PPAR-δ and PPAR-γ were detected throughout 6–48 h following *M*. *leprae* infection (Fig 2B), while dead *M*. *leprae* only transiently boosted the protein expressions of PPAR-δ and PPAR-γ that soon returned to basal levels (Fig 2B). Furthermore, the induction of *PPAR-δ* and *PPAR-γ*, as well as their target genes including *ADRP*, *FABP4*, *CD36*, were notably in a bacterial load-dependent manner (Fig 3A). These results together suggest a committed role of PPAR-δ and PPAR-γ in *M*. *leprae* infection. In addition, a selective PPAR-δ agonist L-165041 induced the gene expressions of *ADRP* and *FABP4* in a dose-dependent manner, reproducing an effect comparable to that of *M*. *leprae* infection at MOI = 100 (Fig 3B).

### Nuclear redistribution of PPAR-δ and PPAR-γ in *M*. *leprae*-infected cells

**Fig 4:**
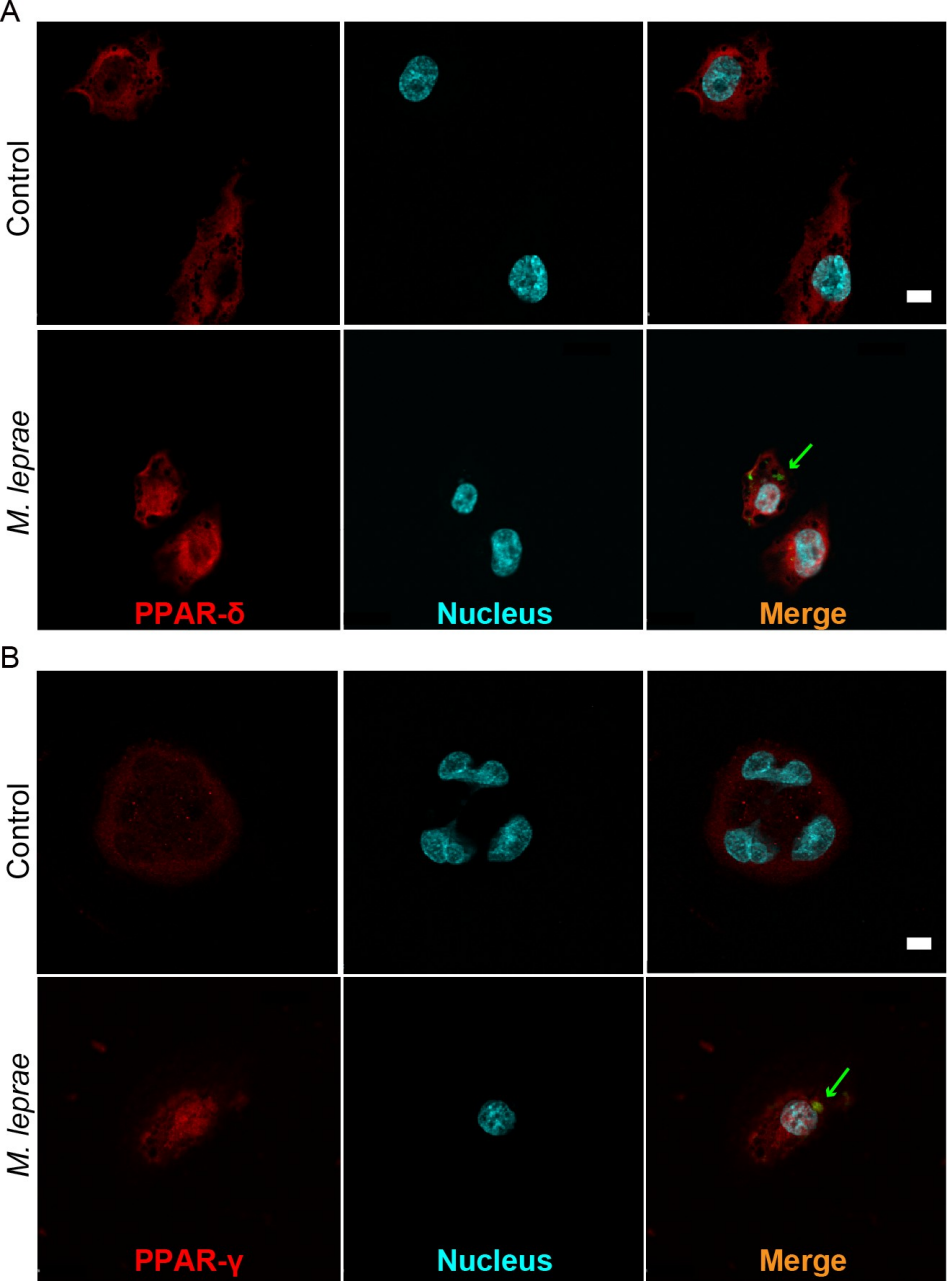
Nuclear translocalization of PPAR-δ and PPAR-γ proteins occur after *M*. *leprae* infection. THP-1 cells grown on glass coverslips in 24-well plates were infected with FITC-conjugated *M*. *leprae* (MOI = 100), indicated by green arrows, for 48 h, followed by immunofluorescence staining for PPAR-δ or PPAR-γ (red). Nuclei were counterstained with Hoechst 33258 (blue). Bars: 10 μm.

The activation of PPARs signaling requires the binding by their ligands, which in turn enables the nuclear translocalization of PPARs [12]. To further access whether *M*. *leprae* infection activates PPAR signaling in host cells, we demonstrated the intracellular distributions of PPAR-δ and PPAR-γ before and after *M*. *leprae* infection by immunofluorescence staining. The results showed that before infection, the nuclear areas were nearly negative for PPAR-δ or PPAR-γ immunostaining, and PPAR-δ and PPAR-γ proteins were mostly detected outside the nuclear areas (Fig 4). By contrast, in *M*. *leprae*-infected cells the PPAR-δ and PPAR-γ proteins were more overlapped with the counterstained nucleus (Fig 4), suggesting that potential nuclear translocalization of PPAR-δ and PPAR-γ, as a hallmark of PPAR signaling activation, likely occurred following *M*. *leprae* infection in host cells.

### Interference with PPAR-δ or PPAR-γ signaling inhibited *M*. *leprae*-inducible host gene expressions and foam cell formation in THP-1 cells

**Fig 5:**
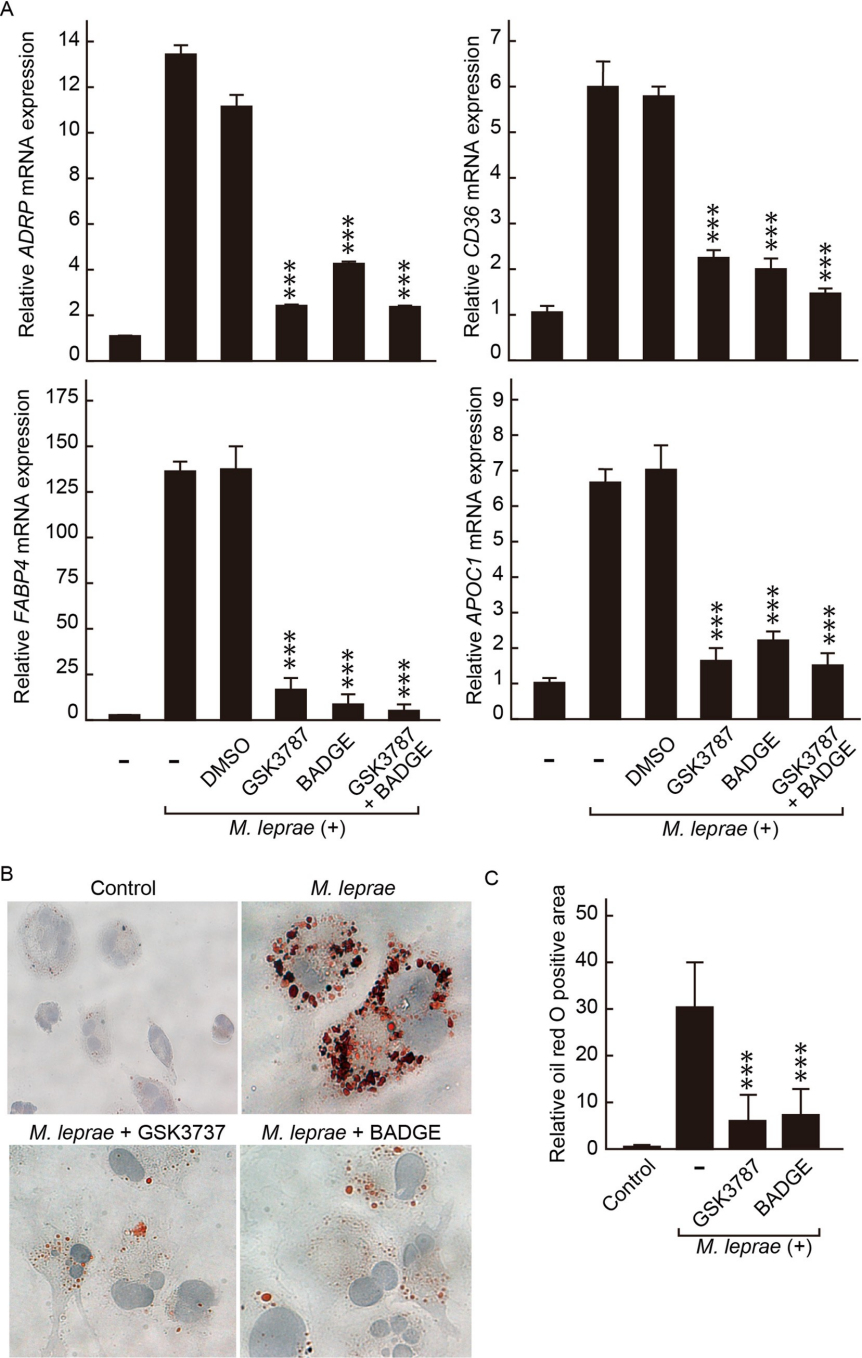
Antagonist of PPAR-δ or PPAR-γ inhibited *M*. *leprae*-induced expressions of adipocyte differentiation-associated genes and foam cell transformation in THP-1 cells. THP-1 cells were infected with *M*. *leprae* MOI (100), in the presence of 0.01% DMSO, 1 μM GSK3787, 10 μM BADGE, or 1 μM GSK3787 and 10 μM BADGE in combination, for 48 h. (A) Total RNAs were extracted, relative mRNA levels of *ADRP*, *FABP4*, *CD36*, and *APOC1* were evaluated by real-time PCR. Data are presented as mean ± SD relative to the control cells (n = 3). ***P < 0.001, compared to cells infected by *M*. *leprae* in the absence of antagonist. (B) THP-1 cells grown on glass coverslips in 24-well plates were infected with *M*. *leprae* MOI (100), in the presence of 1 μM GSK3787 or 10 μM BADGE for 48 h, followed by oil red O staining and hematoxylin counter staining. (C) Oil red O positive area of 100 cells were quantified using ImageJ software and normalized against that of non-infected control cells. Data are presented as mean ± SD relative to the control cells (n > 7). ***P < 0.001, compared to *M*. *leprae-*infected and antagonist-untreated cells.

To investigate whether tampering with PPAR signaling could sabotage the lipid-enriched intracellular environment in host cells, we first examined the adipocyte differentiation-associated gene expressions in *M*. *leprae-*infected cells in the presence of PPAR-δ or/and PPAR-γ antagonists (GSK3787 or/and BADGE). Real-time PCR results showed that either GSK3787 or BADGE alone could reduce *M*. *leprae*-inducible host gene expressions, including *ADRP*, *FABP4*, *CD36*, *APOC1*, to levels similar as that in the control cells (Fig 5A). A combination of GSK3787 and BADGE treatment further reinforced the effect of each antagonist to abolish the effect of *M*. *leprae* infection on host gene expressions (Fig 5A). In accordance, the lipid loads in *M*. *leprae*-infected cells were significantly alleviated by the administration of either GSK3787 or BADGE, as demonstrated by oil red O staining (Fig 5B and 5C). These results together suggest that PPAR signaling is necessary for *M*. *leprae*-induced intracellular accumulation of lipid droplets in host cells.

### ADRP gene expressions in slit-skin smears were specifically diminished after MDT

**Fig 6:**
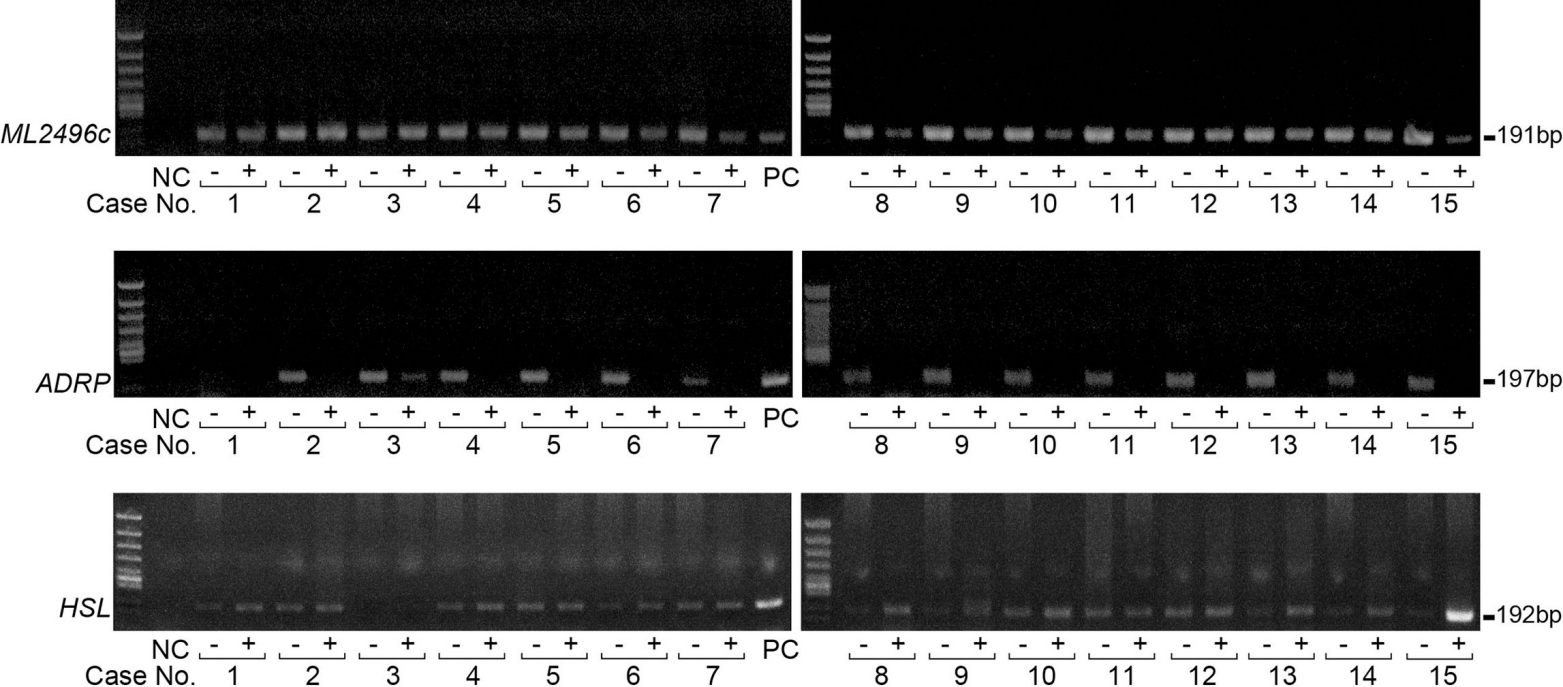
Host ADRP expressions were specifically decreased in skin smear samples after MDT. Total RNAs were extracted from skin smear samples of 15 patients (including lepromatous leprosy: No.3, 5, 9, 10, 14; borderline lepromatous: No. 2, 4, 6, 7, 11, 12, 13, 15; borderline tuberculoid: No. 1; borderline: No. 8) collected before treatment (-) or after the completion of MDT (+). *M*. *leprae*-derived gene *ML2496c*, human *ADRP*, human *HSL* mRNA levels were evaluated by touch-down PCR. NC (negative control): ultrapure H_2_O. PC (positive control) for human *ADRP* was THP-1 cells infected by *M*. *leprae*; PC for human *HSL* was non-infected THP-1 cells.

The above results suggest that PPAR signaling is activated to induce adipocyte differentiation-related genes in *M*. *leprae*-infected macrophages, resulting in accumulation of lipid droplets in host cells. By contrast, dead *M*. *leprae* was unable to induce or sustain the adipocyte differentiation-associated gene expressions, and failed to precipitate foam cell differentiation, indicating that host gene expressions associated with foam cell differentiation specifically respond to active intracellular *M*. *leprae* parasitization. To investigate whether host genes respond to *M*. *leprae* infection similarly *in vivo*, we examined PPAR target gene expressions using RNA extracted from slit-skin smears of patients with leprosy before and after the completion of MDT. *M*. *leprae*-specific gene *ML2496c* whose expressions were detected by RT-PCR in specimens obtained both before and after MDT (Fig 6). Human *ADRP* expressions were detected in 14 clinical samples (including five LL, eight borderline lepromatous (BL), one borderline (BB)) obtained before MDT, except in one specimen derived from borderline tuberculoid (BT) (Fig 6). Intriguingly, host *ADRP* expressions were significantly decreased below detectable levels of touchdown PCR after MDT (Fig 6). Consistently with our previous findings [10], the expressions of host hormone-sensitive lipase (*HSL*), a key molecule in fatty acid mobilization and lipolysis sensitive, were greatly induced in most cases (12/15) after MDT (Fig 6), suggesting that the decreases in *ADRP* expressions were likely a specific outcome of anti-leprosy treatment, but not due to overall lower RNA concentrations. Thus, the *in vivo* results suggest that certain host PPAR target genes potentially serve as innovative biomarkers for active *M*. *leprae* infection in skin lesions.

## Discussion

In this study, we showed that a cluster of PPAR target genes, including *ADRP*, *FABP4*, *CD36*, *APOE*, *APOC1*, *ASCL1*, in parallel with the intracellular lipid accumulation, were significantly increased in *M*. *leprae-*infected cells. PPAR-δ and PPAR-γ gene and protein expressions were induced by *M*. *leprae* infection in a bacterial burden-dependent manner. Immunofluorescence staining suggested that nuclear translocalization of PPAR-δ and PPAR-γ, as a prerequisite step in the activation of PPAR signaling, occurred after *M*. *leprae* infection. Either PPAR-δ or PPAR-γ antagonist, or both in combination, abolished the effect of *M*. *leprae* infection to induce *ADRP*, *FABP4*, *CD36*, *APOC1* expressions in THP-1 cells and also inhibited intracellular lipid accumulation. These results together suggest that in response to *M*. *leprae* infection, host PPAR signaling was activated to induce an array of adipocyte differentiation-associated genes, leading to foam cell differentiation to accommodate *M*. *leprae* parasitization.

PPAR superfamily participates in intracellular lipids metabolisms through transcriptional regulation of genes involved in lipid uptake, transport and storage in adipocytes, monocytes, and macrophages [6], PPAR-γ, perhaps best known as a therapeutic target in treatment for metabolic disorders (such as in diabetes and atherosclerotic) [22,23], primarily functions to increase the storage of intracellular fatty acids and thereby reduces the amount of fatty acids in circulation to improve hyperlipidaemia and hyperglycemia [23]. The role of PPAR-γ in bacterial infection models has also emerged since recently. Up-regulation of PPAR-γ expression in macrophages after infection by *M*. *bovis* bacillus Calmette-Guerin (BCG), *M*. *tuberculosis*, or *Listeria monocytogenes* (*L*. *monocytogenes*) has been reported [7–9,24]. Pretreatment with a PPAR-γ antagonist significantly inhibited BCG/*M*. *tuberculosis*-induced intracellular lipid droplets [8,25,26]. The profile of PPAR-γ target genes are indeed closely involved in lipid droplet biosynthesis: ADRP acts as a nucleation center for the assembly of nascent lipids in macrophages and Schwann cells during mycobacterial infections [11,27]; CD36 assists the uptake and intracellular accumulation of lipids in mycobacteria-infected cells [28–30]; FABP4 transports of fatty acids to facilitates foam cell formation [14,15]; ACSL directly participates in the de novo synthesis of triglyceride from fatty acid within cells [21]. PPAR-δ, although much less studied in the context of mycobacterial infections, shares many target genes with PPAR-γ such as *ADRP*, *FABP4*, *CD36*, *APOE* [6,19,31], and also acts as a regulator in intracellular lipid homeostasis [31]. PPAR-δ gene expression is significantly induced during foam cell differentiation *in vitro*, whereas its activation by selective agonists leads to increased lipids accumulation in primary human macrophages, with increased expressions of *ADRP*, *FABP4*, *CD36* [31,32].

PPAR-γ is also known for an anti-inflammation effect [23,33–35]. PPAR-γ activation during infection by BCG or *M*. *tuberculosis* resulted in an anti-inflammatory response, and suppressed macrophage innate immune functions, whereas PPARγ knockdown in human macrophages led to strengthened macrophage-mediated mycobacterial killing with increased tumor necrosis factor (TNF)-α production and decreased lipid droplet formation [7,36]. Deletion of PPARγ in human alveolar macrophages reduced the growth of virulent *M*. *tuberculosis*, enhanced pro-inflammatory cytokines, and reduced granulomatous infiltration in murine lungs [37,38]. Thus, PPARγ could be crucial for the intracellular growth of mycobacteria through versatile functions in addition to its role in foam cell formation. Whether the immune-regulating effects of PPAR-γ are also involved in *M*. *leprae* infection, and whether PPAR-γ knockdown would augment macrophage-mediated *M*. *leprae* killing, merits further investigations. Comparing to the radical MDT which results in sudden exposure of a large amount dead *M*. *leprae* remnants/antigens risky to trigger an adverse host immune response (i.c. lepra reaction), disintegrating the greasy “fortress” of *M*. *leprae* by inhibiting host PPAR signaling may provide an alternative strategy to treat this disease efficiently and tenderly.

A molecular mechanism by which PPAR-γ is activated upon *M*. *tuberculosis* infection has been proposed: *M*. *tuberculosis* is recognized by mannose receptor (MR) in macrophages, leading to up-regulation of PPAR-γ expression in a MR-dependent manner [7]. Recognition of *M*. *tuberculosis* by MR also activates mitogen-activated protein kinase (MAPK)-p38-cytosolic phospholipase A_2_ (cPLA_2_), resulting in hydrolysis and release of arachidonic acid from the plasma membrane to generate prostaglandin E_2_ (PGE_2_) and cyclopentenone prostaglandins (15-*d*-PGJ_2_) [7]. PGE_2_ and 15-*d*-PGJ_2_ serve as endogenous PPAR-γ ligands to activate PPAR signaling in host macrophages [7]. Whether a similar signaling pathway is employed in *M*. *leprae* infection remains to be investigated.

Last but not least, in contrast to *M*. *leprae* infection, dead *M*. *leprae* failed to sustain host adipocyte differentiation-associated gene expressions, or to induce foam cell formation in macrophages, indicating that host PPAR target genes may serve as potential markers for active *M*. *leprae* infection. In skin smears, *M*. *leprae*-derived gene *ML2496c* was clearly detected both before and after MDT, in consistent with the fact that antibiotics-killed *M*. *leprae* can remain inside the tissues for decades. By contrast, human *ADRP* and *HSL* expression levels could vary dramatically between untreated and MDT-treated cases. Conventionally, BI is the most commonly used test to evaluate the density of bacilli, including both living and dead ones, in lesions. During MDT, it may be found that there is no fall in the BI during the first 12-month. Morphological index (MI), which calculates the percentage of the solid stained (living) (otherwise irregularly-stained deemed as killed bacilli) ones out of 200 fast acid stained bacilli, has been introduced to improve the sensitivity to determine whether infection is active or responding to treatment, and whether the patient has defaulted on treatment or developed bacterial resistance to chemotherapy. However, correct MI heavily relies on wealthy experience in dealing with mycobacterial morphology and Zeihl-Nelson’s staining. Our results suggest that in addition to BI and MI, a test of certain host gene expressions using RNA purified from skin smears (which can be obtained when sampling for BI/MI) may be helpful to improve the sensitivity of monitoring recurrent infection and the treatment efficacy.

## Supporting information

S1 Table Primers used for PCR analysis. (DOCX)

S1 Data Data used to produce the graphs in the figures. (XLSX)
