# Supplementary material for: The function of peroxisome proliferator-activated receptors PPAR-γ and PPAR-δ in Mycobacterium leprae-induced foam cell formation in host macrophages
Source: PLoS Negl Trop Dis. 2020 Oct 19;14(10):e0008850. doi: 10.1371/journal.pntd.0008850 (PMC7595635; doi:10.1371/journal.pntd.0008850)
Supplement: S1 Table — (DOCX) [file pntd.0008850.s001.docx]

**S1 Table. Primers used for PCR analysis.**

| Gene | Primer sequence |
| --- | --- |
| *ADRP* | Forward: 5′-TGTGGAGAAGACCAAGTCTGTG-3′  Reverse: 5′-GCTTCTGAACCAGATCAAATCC-3′ |
| *FABP4* | Forward: 5′-ATGTGCAGAAATGGGATGGAA-3′  Reverse: 5′-CTCGTGGAAGTGACGCCTTT-3′ |
| *CD36* | Forward: 5′-GAGTTTGCAAGAAACAGGTGCT-3′  Reverse: 5′-TCCAAACACAGCCAGGACAG-3′ |
| *ACSL1* | Forward: 5′-CTTTTGCAGCACTCACCACC-3′  Reverse: 5′-CCTGAGCGATCTTGACACCT-3′ |
| *APOE* | Forward: 5′-GCAGACCGAGTGGCAGAG-3′  Reverse: 5′-GTTCCTCCAGTTCCGATTTG-3′ |
| *APOC1* | Forward: 5′-GACAGGAATCTTGCCCATTCC-3′  Reverse: 5′-TTTCCCCACTCAGAATGTAGCA-3′ |
| *ABCA1* | Forward: 5′-AACTCTACATCTCCCTTCCCG-3′  Reverse: 5′-CTCCTGTCGCATGTCACTCC-3′ |
| *PPAR-α* | Forward: 5′-GGCGAGGATAGTTCTGGAAGC-3′  Reverse: 5′-CACAGGATAAGTCACCGAGGAG-3′ |
| *PPAR-δ* | Forward: 5′-TGTCTCCCTCTTTCTCAGTT-3′  Reverse: 5′-AGGGAAGAGAGAAACCTACA-3′ |
| *PPAR-γ* | Forward: 5′-CACAAGAACAGATCCAGTGGTTGCAG-3′  Reverse: 5′-AATAATAAGGTGGAGATGCAGGCTCC-3′ |
| *ML2496* | Forward: 5′-ACTAGCGGTATCGATCTGAC-3′  Reverse: 5′-GTGATGCGTTGGAATTCGG-3′ |
| *β-ACTIN* | Forward: 5′-AGCCATGTACGTAGCCATCCT-3′  Reverse: 5′-TGTGGTGGTGAAGCTGTAGC-3′ |
